# Impact of the Coronavirus Disease Pandemic and Related Vaccination in an Orthopedic Clinic in the United Arab Emirates: An Observational Study

**DOI:** 10.3389/fsurg.2022.906797

**Published:** 2022-05-31

**Authors:** Seung-Kook Kim, Seo-jung Park, Dae-won Cho, Hong-suk Kwak, Hee-yon Jin, Su-hyun Eum, Eun-jung Heo, Gi-eun Kim, Ha-young Ji, Seung-jun Park

**Affiliations:** ^1^ Himchan UHS Spine and Joint Centre, Neurosurgery, University Hospital Sharjah, University City 1, Sharjah, United Arab Emirates; ^2^ Joint and Arthritis Research, Orthopaedic Surgery, Himchan Hospital, Seoul, Korea; ^3^ Department of Pharmaceutical Medicine and Regulatory Sciences, College of Medicine and Pharmacy, Yonsei University, Seoul, Korea; ^4^ Himchan UHS Spine and Joint Centre, Orthopedic Surgery, University Hospital Sharjah, Sharjah, University City 1, Sharjah, United Arab Emirates; ^5^ Himchan UHS Spine and Joint Centre, Physiotherapy, University Hospital Sharjah, University City 1, Sharjah, United Arab Emirates

**Keywords:** COVID-19, coronavirus, pandemic, vaccination, orthopedics

## Abstract

The coronavirus disease (COVID-19) pandemic has influenced hospital visiting patterns. Although vaccination has decreased infection rates and disease severity, hospital visiting patterns and associated treatment changes related to orthopedics remain unexplored in the Middle East. Therefore, this study aimed to examine the impact of the COVID-19 pandemic and vaccination on individual departments dealing with musculoskeletal disorders in the United Arab Emirates. Relationships between publicly available national data on the number of COVID-19 polymerase chain reaction tests and confirmed and recovered cases during May 2020–July 2021 and hospital data on the number of outpatients, inpatients, operations, and physiotherapy consultations were analyzed. In January 2021, the relationship between vaccination rate and orthopedic unit utilization was evaluated after vaccination campaign initiation. Multifactorial analysis revealed that an increased number of COVID-19-related deaths correlated with a decreased number of joint operations. Negative linear relationships were observed among confirmed and death cases with inpatient treatment and joint operation as well as recovered cases with inpatient treatment. Recovered cases with inpatient treatment and joint operation showed a positive linear relationship. Inpatient spine treatment showed a positive relationship with vaccination rates. The COVID-19 pandemic influenced orthopedic treatment in the Middle East, and vaccination campaigns facilitated inpatient spine treatment.

## Introduction

The coronavirus disease (COVID-19) pandemic has affected individuals’ social behaviors, including the frequency of hospital visits. There has been a significant decrease in the number of hospital treatments delivered across specialties (except those managing COVID-19 cases), especially in the emergency and outpatient departments (1, 2). Although this trend has been observed for all in-hospital treatments, nonvital departments have been less affected than vital departments (3). Orthopedic treatments, especially those related to the spine, have been shown to improve patient quality of life. Furthermore, failure of timely treatment can be detrimental and decrease survival rates (4). After the large-scale implementation of vaccination and emergent approval for vaccination by the World Health Organization, it is expected that individuals’ social lives will normalize, and the pattern of hospital visits will improve before the pandemic ends. However, the pandemic and vaccination impacts on the orthopedic field in Middle Eastern countries have not yet been reported. This study aimed to evaluate the effects of the COVID-19 pandemic and the vaccination campaign in the United Arab Emirates (UAE) on the different types of orthopedic treatments being delivered.

## Materials and Methods

This was an observational study related to the effects of the COVID-19 pandemic (May 2020 to July 2021) at a single center specializing in spine and joint treatments at a tertiary hospital (Himchan Spine and Joint Center, University Hospital Sharjah) in the UAE. This study was approved by the Institutional Review Board of University Hospital Sharjah (approval no: UHS-HERC-077-15112021). The vaccination campaign analysis was initiated after approval of the Ministry of Health of the UAE (January to July 2021). The hospital data related to the type of treatment delivered (outpatient, inpatient, operation, and physiotherapy) were managed using a computerized system and organized by the administration team (S. Park and H. Jin). The term “operation” in this study corresponded to a major operation in the operating theater and did not include percutaneous injection procedures in the outpatient clinic. All physician therapists and nurses always wore N-95 masks during outpatient treatment and surgery. Faceguards were used throughout treatment from May to October 2020; however, after that period, a mask was worn before and after every procedure, and handwashing was done. In the spine department, the main treatment methods were diagnosis, injection in the outpatient clinic, laminectomy and fusion surgery. Degenerative disease, trauma injection and conservative treatment were done in the outpatient section of the joint department, and surgery was mainly for fracture, arthroscopy, and joint replacement. Open data related to COVID-19 tests confirmed and recovered cases, deaths, and vaccination rates in the UAE are used by the National Emergency Crisis and Disaster Management Authority (NCEMA) to inform the general public. The open data have been used to acquire information on the polymerase chain reaction (PCR) tests conducted and determine the monthly count of the numbers of confirmed, recovered, and death cases. All data strictly followed the open data policy in NCEMA, and personal data were not used in this study. Data regarding in-hospital visits included outpatient visits, inpatient admissions, and operation counts in each department of the spine, joint, and physiotherapy center. From May to July 2020, the hospital used 1–2 wards for corona treatment, admission beds were decreased, and non-urgent operations were recommended to be delayed. Our infection control committee strictly controlled the preventive methods for Covid-19 infection. Body temperature and a questionnaire for related symptoms were evaluated in the outpatient clinic and physiotherapy. Visitors were not allowed without being accompanied by one guardian. N-95 Mask use and hand hygiene were mandatory for our staff. Vaccination campaigns started in the UAE in January 2021. Three vaccines are currently available: BBIBP-Corv (Sinopharm, Beijing CNBG, Beijing, China), Pfizer-BioNTech (Pfizer, Inc., Philadelphia, PA, USA), and Sputnik V COVID-19 vaccine (Gam-COVID-Vac, Gamaleya Research Institute of Epidemiology and Microbiology, Moscow, Russia).

Statistical analyses were performed using the R software for Windows (version 3.6.0, Vienna, Austria). The factors affecting the patient count at hospitals were analyzed using multiple regression analysis. Correlation analysis between two variables was performed using linear analysis with Pearson’s correlation coefficient (Pearson’s r). Statistical significance was set at *p*-values <0.05.

## Results

The national data on COVID-19 cases and hospital utilization are summarized in Table 1.

**Table 1:** Patient number (outpatient and inpatient), operation count, and physiotherapy consultation during the COVID-19 pandemic in the United Arab Emirates.

	May 2020	Jun 2020	Jul 2020	Aug 2020	Sep 2020	Oct 2020	Nov 2020	Dec 2020	Jan 2021	Feb 2021	Mar 2021	Apr 2021	May 2021	Jun 2021	Jul 2021
Daily tests	1,089,866	1,357,576	1,483,576	2,018,274	2,610,068	3,479,434	3,543,581	4,162,600	4,874,379	4,901,131	6,933,197	6,509,867	6,230,067	7,396,685	8,186,876
Recovered cases	15,503	19,635	16,434	7,022	22,793	45,178	25,997	29,543	92,516	104,267	64,130	55,424	49,746	60,917	46,756
Deaths	159	51	36	33	35	76	77	97	181	371	276	90	93	131	138
Outpatients (Spine)	243	499	655	688	783	665	751	631	688	557	708	538	583	697	407
Outpatients (Joint)	246	492	631	700	772	645	650	768	599	618	730	558	490	673	549
Inpatients (Spine)	25	56	57	86	75	117	65	78	18	11	47	35	36	75	54
Inpatients (Joint)	34	93	125	104	112	85	92	96	18	25	4	40	18	157	96
Operations (Spine)	7	22	19	24	24	29	16	22	16	11	30	18	14	25	18
Operations (Joint)	9	15	21	23	22	21	20	18	9	6	1	11	12	18	12
Physiotherapy consultations	455	1,044	1,119	1,165	1,307	1,302	1,389	1,572	1,217	1,216	1,353	1,106	957	1,423	1,184
Vaccination (%)	NA	NA	NA	NA	NA	NA	NA	NA	33.71	60.87	84.1	106.64	130.13	154.51	169.82

*COVID-19, coronavirus disease; NA, not available.*

### COVID-19 PCR Test, Confirmed Cases, and Orthopedic Treatment

**Figure 1:**
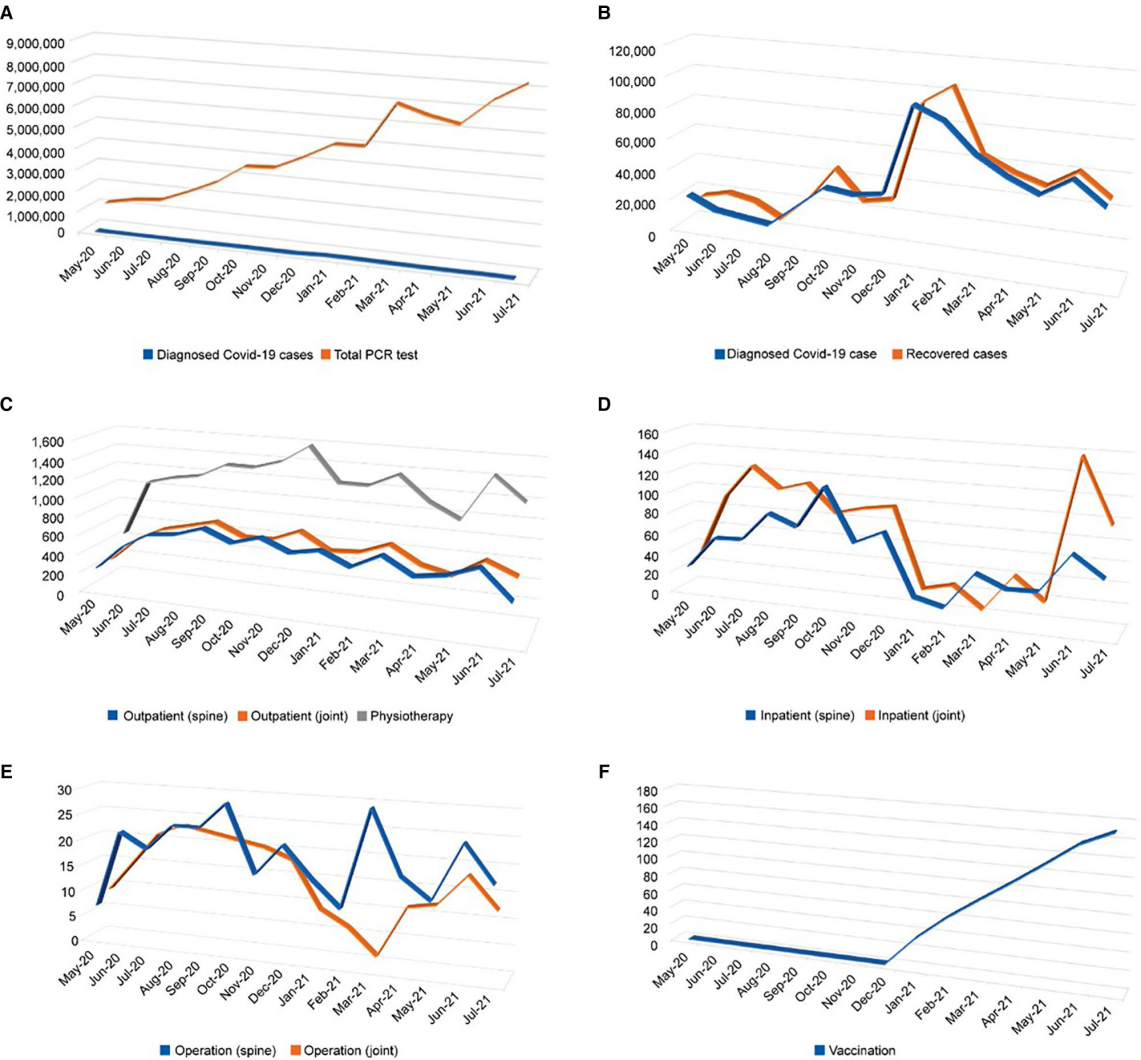
Graphs of national data of coronavirus disease 2019 (COVID-19) and hospital visiting patterns. (**A**) Diagnosed cases and number of polymerase chain reaction (PCR) tests; (**B**) Diagnosed and recovered cases; (**C**) Outpatient count in each department; (**D**) Inpatient count in each department; (**E**) Operation count in each department; (**F**) Vaccination rate.

The number of PCR tests conducted showed an increasing trend until July 2021, with a monthly count reaching approximately 8 million cases (Figure 1A). The number of confirmed cases peaked in January 2021 at approximately 80,000 cases/month and decreased spontaneously to 40,000 cases /month in July 2021. Recovered cases peaked at 1,000,000/month in February 2021 and showed a decreasing trend similar to that of the confirmed cases (Figure 1B). After the number of confirmed COVID-19 cases increased in January 2021, outpatient counts in the joint and physiotherapy departments decreased spontaneously until July 2021 (Figure 1C). The spine department showed a continuous outpatient count of approximately 600 cases per month compared with other departments. Patient counts fluctuated more in the joint department than in that of the spine after the peak duration (from January 2020 to March 2021), i.e., the number of reported inpatient cases increased dramatically to 140 cases per month (Figure 1D). Furthermore, joint operations varied more than spine operations; they decreased until February 2021 and peaked in March 2021. Spine operations showed stable counts, except in March 2021 (Figure 1E). The vaccination rate in the UAE, after the campaign initiation in December 2020, increased monthly and approached an accumulated rate of 169.8% in July 2021 (Figure 1F).

### Multiple Regression Analysis Considering COVID-19 and the Use of Orthopedic Treatments

The influences of various parameters related to COVID-19 on orthopedic department utilization are summarized in Table 2. Outpatient counts across departments, including those related to the spine, joints, and physiotherapy, were not affected. Regarding inpatient treatment, diagnosis in the joint department (*p* = 0.2341) and death count in both the departments (spine: *p* = 0.2689; joint: *p* = 0.2409) had negative relationships with the increase in COVID-19 cases, but these differences were not significant. The operation count in the joint department was only affected by the death count in all multiple regression analyses (*p* = 0.0112).

**Table 2:** Multiple regression analysis regarding COVID-19 in our orthopedic clinic in the United Arab Emirates.

	Outpatient count						Inpatient count				Operation count			
	Spine		Joint		Physiotherapy		Spine		Joint		Spine		Joint	
	Coefficient (95% CI)	*p*-value	Coefficient (95% CI)	*p*-value	Coefficient (95% CI)	*p*-value	Coefficient (95% CI)	*p*-value	Coefficient (95% CI)	*p*-value	Coefficient (95% CI)	*p*-value	Coefficient (95% CI)	*p*-value
Total number of PCR tests	−1.123 × 10^−05^ (6.721 × 10^−05^, 4.475 × 10^−05^)	0.6644	8.927 × 10^−06^ (−4.769 × 10^−05^, 6.554 × 10^−05^)	0.7326	2.952 × 10^−05^ (−7.237 × 10^−05^, 3.554 × 10^−05)^	0.5331	3.586 × 10^−06^ (−5.937 × 10^−06^, 1.311 × 10^−05^)	0.4211	6.526 × 10^−06^ (−8.336 × 10^−06^, 2.139 × 10^−05^)	0.3510	1.136 × 10^−06^ (−1.454 × 10^−06^, 3.726 × 10^−06^)	0.3514	−2.676 × 10^−07^ (1.800 × 10^−06^, 1.265 × 10^−06^)	0.7053
Confirmed cases	0.0038 (−0.0119, 0.0196)	0.6001	0.0005 (−0.0155, 0.0164)	0.9507	0.0026 (−0.0261, 0.0313)	0.8427	−0.0009 (−0.0036, 0.0017)	0.4564	−0.0024 (−0.0066, 0.0018)	0.2341	−0.0002 (−0.0009, 0.0006)	0.6420	−0.0002 (−0.0006, 0.0003)	0.3756
Recovered cases	0.0003 (−0.0138, 0.0145)	0.1623	0.0008 (−0.0135, 0.0151)	0.9005	0.0011 (−0.0246, 020268)	0.9243	0.0005 (−0.0019, 0.0029)	0.6427	0.0016 (−0.0022, 0.0053)	0.3659	0.0001 (−0.0005, 0.0008)	0.7114	0.0002 (−0.0002, 0.0006)	0.311
Death cases	−1.0632 (−2.6332, −0.5069)	0.9576	−0.4814 (−2.0693, 1.1064)	0.5146	−1.12 (−3.9831, 1.7322)	0.4008	−0.1403 (−0.4074, 0.1268)	0.2689	−0.2332 (−0.65, 0.1836)	0.2409	−0.0222 (−0.0948, 0.0505)	0.5123	−0.0598 (−0.1028, −0.0169)	0.0112*

*COVID-19, coronavirus disease; CI, confidence interval; PCR, polymerase chain reaction.*

* *p-values <0.05 are considered statistically significant.*

### Linear Relationships Between PCR Tests and Use of Orthopedic Treatments

The relationships between COVID-19 and orthopedic department utilization are summarized in Table 3.

**Table 3:** Pearson’s correlation of COVID-19 counts and orthopedic clinics in the United Arab Emirates.

	Total tests		Confirmed cases		Recovered cases		Death cases		Vaccination count per population	
	Pearson’s correlation value (r)	*p*-value	Pearson’s correlation value (r)	*p*-value	Pearson’s correlation value (r)	*p*-value	Pearson’s correlation value (r)	*p*-value	Pearson’s correlation value (r)	*p*-value
Outpatient number										
Spine	0.063	0.8231	0.115	0.6839	0.09	0.7505	−0.18	0.5213	−0.444	0.3185
Joint	0.188	0.5020	0.109	0.6981	0.092	0.7431	−0.067	0.8133	−0.58	0.1721
Physiotherapy	0.365	0.1813	0.264	0.3409	0.231	0.4064	0.007	0.9808	−0.442	0.3232
Inpatient number										
Spine	−0.186	0.5065	−0.527	0.0435*	−0.519	0.0476*	−0.607	0.0165*	0.164	0.7253
Joint	−0.208	0.4572	−0.554	0.0321*	−0.521	0.0466*	−0.612	0.0152*	0.35	0.4411
Operation										
Spine	0.136	0.6287	−0.132	0.6383	−0.135	0.6309	−0.261	0.3482	0.141	0.7628
Joint	−0.489	0.085	−0.676	0.0057*	−0.648	0.0090*	−0.823	0.0002*	0.098	0.8337

*COVID-19, coronavirus disease.*

* *p-values <0.05 are considered statistically significant.*

**Figure 2:**
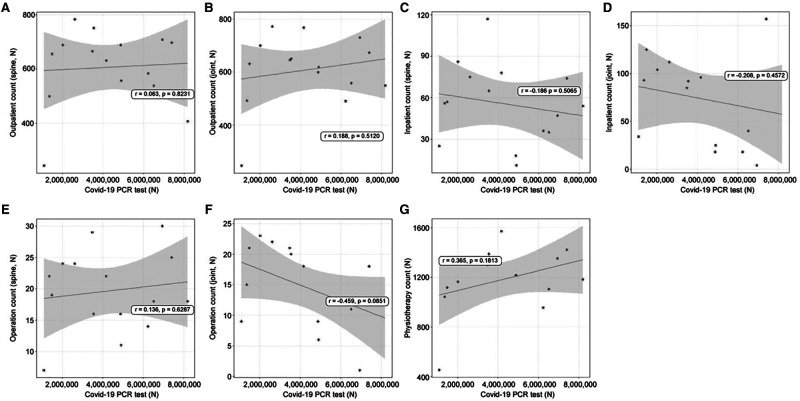
The linear relationships between coronavirus disease 2019 (COVID-19) polymerase chain reaction (PCR) test count and orthopedic hospital visiting patterns. (**A**) Spine department outpatient count; (**B**) Joint department outpatient count; (**C)** Spine department inpatient count; (**D**) Joint department inpatient count; (**E**) Spine operation patient count; (**F**) Joint operation patient count; (**G**) Physiotherapy patient count.

As PCR testing increased, outpatient treatment increased (spine: *r* = 0.063, joint: *r* = 0.188, Figures 2A, B) and inpatient treatment decreased (spine: *r* = −0.186, joint: *r* = −0.208, Figures 2C, D). In addition, operations in the spine department increased slightly (*r* = 0.136, Figure 2E), but those in the joint department decreased (*r* = −0.489, Figure 2F). Physiotherapy patient counts increased (*r* = 0.264, Figure 2G); however, these increases and decreases were not significant.

### Linear Relationships Between Confirmed Cases and the Use of Orthopedic Treatments

**Figure 3:**
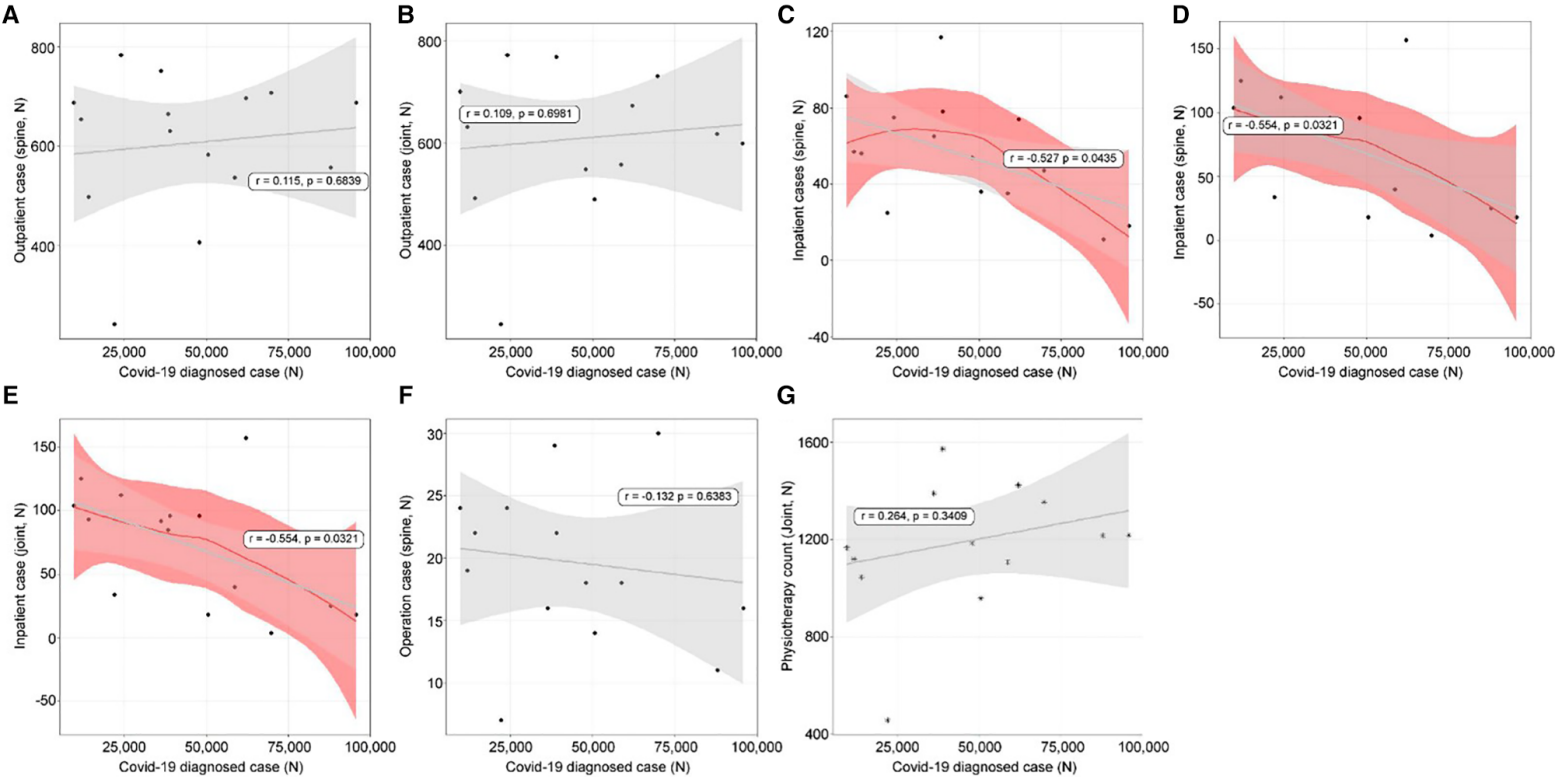
The linear relationships between coronavirus disease 2019 (COVID-19) diagnosis and orthopedic hospital visiting patterns. (**A**) Spine outpatient count; (**B**) Joint outpatient count; (**C**) Spine inpatient count; (**D**) Joint inpatient count; (**E**) Spine operation patient count; (**F**) Joint operation patient count; (**G**) Physiotherapy patient count.

With increasing diagnoses, outpatient treatment increased, but not significantly, in both the spine (*r* = 0.115, *p* = 0.6839, Figure 3A) and joint departments (*r* = 0.109, *p* = 0.6981, Figure 3B). However, inpatient treatment decreased in both the departments; the joint department showed a more significant decline (spine: *r* = −0.527, *p* = 0.0423, Figure 2C; Joint; *r* = −0.554, *p* = 0.0321, Figure 3D). Concerning operations, those of the spine (*r* = −0.132, *p* = 0.6383, Figure 2E) and joints (*r* = −0.459, *p* = 0.0851) decreased, but the differences were not significant. Physiotherapy patient counts showed an insignificant relationship (*r* = 0.264, *p* = 0.3409, Figure 3G).

### Linear Relationships Between Recovered Cases and Use of Orthopedic Treatments

**Figure 4:**
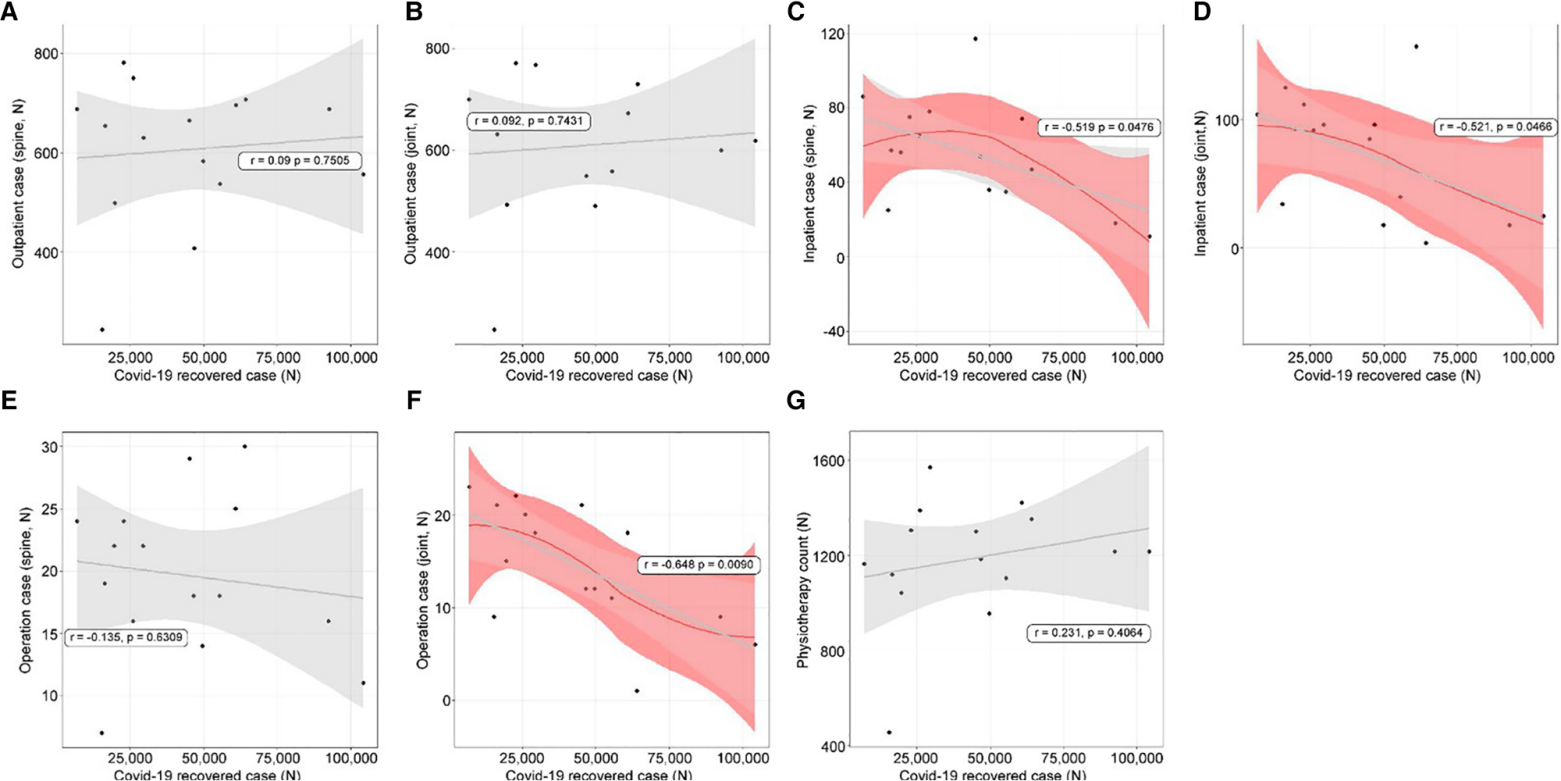
The linear relationships between cases that recovered from coronavirus disease 2019 (COVID-19) and orthopedic hospital visiting patterns. (**A**) Spine outpatient count; (**B**) Joint outpatient count; (**C**) Spine inpatient count; (**D**) Joint inpatient count; (**E**) Spine operation patient count; (**F**) Joint operation patient count; (**G**) Physiotherapy patient count.

Recovered cases and the use of orthopedic treatments were not strongly related. Outpatient treatments (spine: *r* = 0.09, *p* = 0.7505, Figure 4A; joint: *r* = 0.092, *p* = 0.7431, Figure 4B) had insignificant decreases. Inpatient treatments (spine: *r* = −0.519, *p* = 0.0476, Figure 4C; joint: *r* = −0.521, *p* = 0.0466, Figure 4D) had significant decreases. Spine operation patient counts showed no relationship (*r* = −0.135, *p* = 0.6309, Figure 4E); however, those of the joints (*r* = −0.648, *p* = 0.0090, Figure 4F) showed a negative relationship. Physiotherapy patient counts showed an insignificant relationship (*r* = 0.231, *p* = 0.4064, Figure 4G).

### Linear Relationships Between Death Cases and Use of Orthopedic Treatments

**Figure 5:**
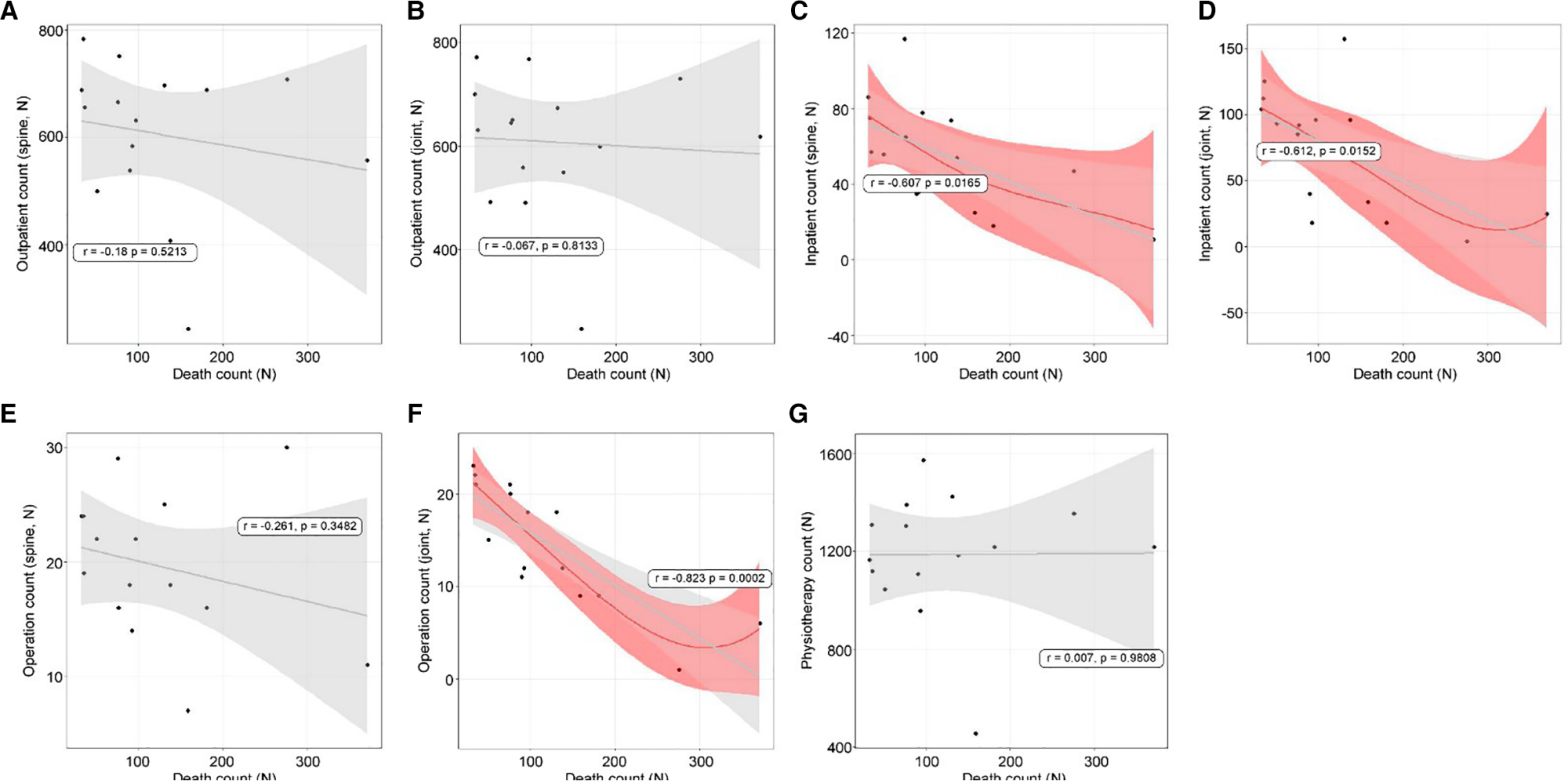
The linear relationships between death counts and orthopedic hospital visiting patterns. (**A**) Spine outpatient count; (**B**) Joint outpatient count; (**C**) Spine inpatient count; (**D**) Joint inpatient count; (**E**) Spine operation patient count; (**F**) Joint operation patient count; (**G**) Physiotherapy patient count.

Death cases and the need for orthopedic treatments affect inpatient treatments and joint operations. Outpatient treatments (spine: *r* = −0.18, *p* = 0.5213, Figure 5A; joint: *r* = −0.067, *p* = 0.8133, Figure 5b) had insignificant decreases. Inpatient treatments (spine: *r* = −0.607, *p* = 0.0165, Figure 5C; joint: *r* = −0.612, *p* = 0.0152, Figure 5D) had significant decreases. Spine operation patient counts (*r* = −0.261, *p* = 0.3482, Figure 5E) showed no relationship; however, joint operation patient counts (*r* = −0.823, *p* = 0.0002, Figure 5F) showed a negative relationship. Physiotherapy patient counts showed an insignificant relationship (*r* = 0.007, *p* = 0.9808, Figure 5G).

### Linear Relationships Between Vaccination Rate and Use of Orthopedic Treatments

**Figure 6:**
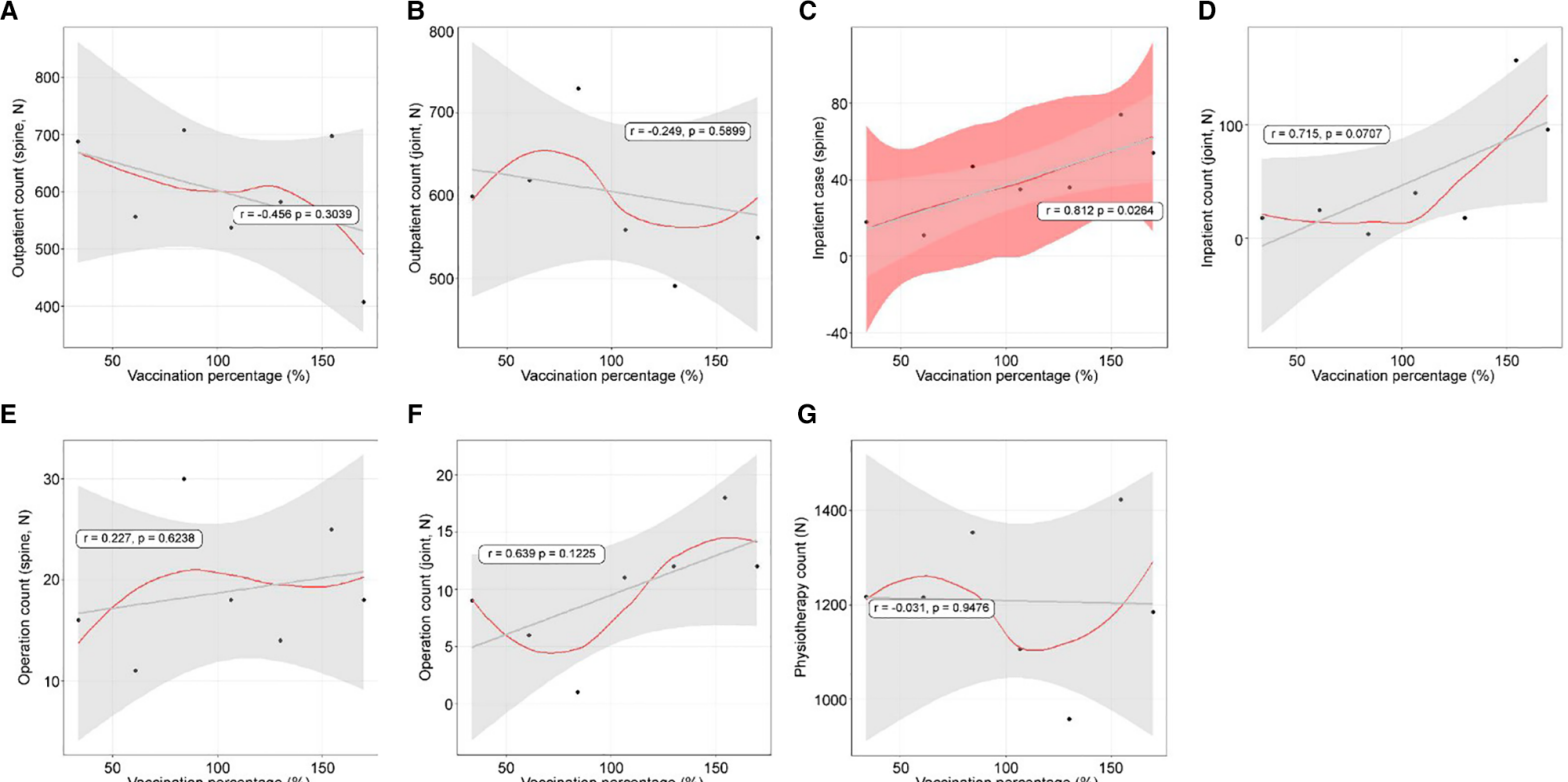
The linear relationships between vaccination rate and orthopedic hospital visiting patterns. (**A**) Spine outpatient count; (**B**) Joint outpatient count; (**C**) Spine inpatient count; (**D**) Joint inpatient count; (**E**) Spine operation patient count; (**F**) Joint operation patient count; (**G**) Physiotherapy patient count.

The linear relationships between the vaccination rate and use of orthopedic treatments are summarized in Table 4. Outpatient treatments (spine: *r* = −0.456, *p* = 0.3039, Figure 6A; joint: *r* = −0.249, *p* = 0.5899, Figure 6B) had insignificant decreases. Inpatient spine treatments showed a linear increase (*r* = 0.812, *p* = 0.264, Figure 6C). Inpatient joint treatments increased, but this was not significant (*r* = 0.715, *p* = 0.0707, Figure 6D). The number of operations conducted in the spine department (*r* = 0.227, *p* = 0.6238, Figure 6E) and joint department (*r* = 0.639, *p* = 0.1225, Figure 6F) did not show any relationships. Physiotherapy patient counts showed an insignificant relationship (*r* = 0.031, *p* = 0.9476, Figure 6G).

**Table 4:** Linear relationship between the vaccination rate and visits to the orthopedic clinic.

	Pearson’s correlation value	*p*-value
Outpatient		
Spine	−0.4559	0.3039
Joint	−0.2492	0.5899
Physiotherapy	−0.0309	0.9476
Inpatient		
Spine	0.8125	0.0264*
Joint	0.715	0.0707
Operation		
Spine	0.2274	0.6238
Joint	0.6389	0.1225

*p-values < 0.05 are considered statistically significant.

## Discussion

This study demonstrated that our hypotheses that the COVID-19 pandemic would decrease the number of outpatients, inpatients, and operations and that these numbers would normalize with vaccination coverage were partially correct. Regarding the multifactor relationship, the number of joint operations decreased; however, upon evaluation of single-factor relationships, linear correlations were observed between COVID-19 diagnosis and both inpatient count and number of operations in the joint department. The recovered cases with inpatient treatment and death cases with both inpatient treatment and operations in the joint department showed a relationship. With the increasing vaccination rate, the number of spine operations increased, but that of joint operations did not.

Confirmed and recovered cases showed the same tendency; therefore, these factors can be evaluated together (5). Many factors influence hospital visits for outpatient treatment: “pandemic fever,” or the fear of visiting the hospital, was the main reason highlighted in a previous study (6). Government-imposed lockdowns may be another factor (7). Additionally, the popularity of telemedicine has considerably decreased outpatient treatments (8). Interestingly, the spine, joint, and physiotherapy departments showed no significant decrease in the number of outpatients in this study; this can be explained by the treatments delivered at the orthopedic clinic. Conditions in this field primarily include fractures, sprains, and herniated intervertebral discs that require direct intervention. For example, splint placement, all types of injection administrations, and physiotherapy require physical contact and specialized equipment. In addition, chronic pain management in elderly patients warrants regular visits and physiotherapy as needed. However, inpatient treatment showed a linear decrease in the number of confirmed and recovered COVID-19 cases owing to the lack of available beds. In the UAE, the peak of confirmed COVID-19 cases was during January and February 2021, and many beds were occupied by respiratory disease patients, mainly those with COVID-19. Bed unavailability is unavoidable; however, it should be considered that orthopedic patients requiring inpatient treatment may have to pay the price. Operation count can be affected during a pandemic due to a lack of blood for transfusions and bed unavailability (9). We found that joint operations decreased significantly compared to spine operations, likely because the timing of surgery is more crucial in spine operations (10). In addition, considering the urgency of the situation, spine surgery can be prioritized in a limited bed situation (11).

The number of PCR-confirmed COVID-19 active cases is controversial, and this may be a reason for the temporary increase in the number of confirmed cases (12). In the UAE, an increase in the number of confirmed cases was reported in January 2021, which gradually decreased within a year (13). The field of orthopedics also recovered early with the detection of asymptomatic infections. Although the number of PCR tests has increased and the diagnosis and death rates have decreased, orthopedic treatments remained unaffected. Reportedly, COVID-19 death rates can affect one’s psychological health (14, 15); this could be a reason for the decreased number of joint operations due to staying or admission to the hospital. Limited bed availability with increased numbers of COVID-19 inpatients could be another reason for this decrease. The bed availability rate decreased for other patients due to longer hospitalization periods (16) and difficulty in treatment (17) of patients with COVID-19. One study reported higher mortality and associated complications in COVID-19-positive patients following hip fracture treatment (18). It also reported that the same orthopedic treatment is more challenging in a COVID-19-positive patient than in a non-COVID-19 patient.

Owing to the pandemic, the utilization of hospital services has changed. Outpatient consultations at the departments of internal medicine and familial medicine decreased by 14% and those at the dental clinic by 7% in East Asian countries (19). Hospital visits decreased due to government-imposed movement restrictions depending on the country (20). Pediatric departments also reported a decreased inflow of patients, though only the overall decline has been confirmed owing to differences in hospital policies globally (21). In the UAE, there were no restrictions on visiting and practicing in hospitals during the pandemic, yet our inpatient treatment was affected, and the number of joint operations decreased. We also experienced a lack of inpatient beds due to the increasing number of patients in the internal medicine department. In addition, medical supplies from other departments were diverted to care for patients with COVID-19. We believe that these factors contributed to a decrease in inpatient treatment. We believe a decrease in joint operations compared to spine operations was observed due to the severity of spinal cord injuries and neurological emergencies. Despite the surgery being the same, hospital admission was decreased, and non-urgent surgery was delayed. The number of spine operations normalized earlier than that of joint operations following sufficient vaccination coverage for the same reasons.

The efficacy of COVID-19 vaccination remains controversial; other strains (delta and gamma) are decreasing the efficacy (22). However, vaccination imparts direct protection to elderly patients and patients with comorbidities (23). To achieve herd immunity, most of the population should be vaccinated (24). The vaccination rate can vary across regions and cultures (25). According to our results, with >160% vaccination coverage, patients who underwent spine operations have recovered despite the ongoing pandemic. Although this result is not overestimated, it cannot be the result of vaccination alone. Social distancing and mask use have already shown their benefits (26). All preventive measures have a role in the era of COVID-19 (27). The adoption of these practices could help in normalizing hospital treatments. Our outpatient treatments did not decrease during the pandemic; only inpatient treatments decreased due to other departments’ situations. The number of confirmed cases and deaths has drastically decreased following the strict implementation of face masks and vaccination. Nonetheless, psychological recovery is the main factor for the normalization of hospital visiting patterns. Based on our results, the joint department is more effective during the corona pandemic than the spine department, and the vaccination rate affects the normalization of clinic treatment. During a pandemic, prevention methods including wearing a mask, hand hygiene and social distancing, vaccination and prediction of patient pattern change should be observed.

This study has some limitations. Each vaccine has different efficacies; however, these were not evaluated. Our results would be more reliable if each vaccine’s efficacy were analyzed. Our results cannot be generalized to other medical fields. The orthopedic department deals mainly with outpatients and involves many interventions. Other areas with different therapeutic characteristics may have different results. Further studies are required to compare the results in each field. Even within the same clinic, various diagnoses and different treatments are required. The current study focused on general trend changes in orthopedic clinics; however, future studies will be valuable to evaluate each disease separately.

In conclusion, in the UAE, orthopedic treatment was influenced by the COVID-19 pandemic, including patient diagnosis, recovery, and death. With the increasing vaccination rate, the treatment pattern has seemed to normalize.

## Data Availability

The original contributions presented in the study are included in the article/Supplementary Material, further inquiries can be directed to the corresponding author/s.
